# Editorial and Publishing Arrangements

**Published:** 1835

**Authors:** 


### II.--ORIGINAL.

### The Western Journal.

*E Sylvis Nuncius.*

CINCINNATI, JULY 1, 1835.

### I. — EDITORIAL AND PUBLISHING ARRANGEMENTS.

AGREEABLY to a notice in our last number, the subscription list of the Western Medical Gazette has been united with that of the Western Journal, which will be sent to the subscribers of the former, who, unless they return this number, will be regarded as subscribers to the consolidated Journal.

It will be seen that the publishers, Dr. Charles R. Cooper and Dr. Silas Reed, are also, assistant editors. The articles which they contribute will be marked with the initials of their respective names; those not thus indicated will be from the pen of the senior editor.

